# A SCOPING REVIEW OF OBESITY INTERVENTIONS AND EPIDEMIOLOGICAL STUDIES AMONG OLDER RURAL ADULTS

**DOI:** 10.1093/geroni/igae098.3778

**Published:** 2024-12-31

**Authors:** Maki Karakida, Jaqueline Avila

**Affiliations:** University of Massachusetts Boston, Boston, Massachusetts, United States; University of Massachusetts Boston, Boston, Massachusetts, United States

## Abstract

Rural communities are known for their limited access to care, transportation, and healthy foods, which can increase obesity risks. Obesity among older adults is associated with mobility impairment, falls, diabetes, hypertension, and can further deteriorate the health of older rural residents. This scoping review assessed studies targeting obesity/overweight among rural adults aged 50+ to: 1) analyze the prevalence of obesity and the highest risk groups in epidemiological studies, and 2) assess the efficacy of clinical/community interventions to reduce obesity/overweight in this population. CINAHL Ultimate, PubMed, Google Scholar, and MEDLINE databases were searched from January 1, 2000 to June 27, 2024. Fifteen articles met the eligibility criteria. Selected studies were then divided into: community interventions (n= 3), remote interventions (n= 3), hybrid intervention (n= 1), and cross-sectional epidemiological studies in the US (n= 3) and abroad (n= 5). Epidemiological studies consistently showed that the prevalence of obesity/overweight was higher among older women than men. While one US study found significant associations between lower educational attainment and higher obesity rates, one non-US study reported no associations between obesity and educational levels. Community interventions included nutrition and exercise sessions. One randomized-controlled intervention significantly reduced participant’s weight compared to those who did not receive face-to-face counselling. Remote/hybrid interventions, including telehealth and video conferencing, were cost- and time-effective, but only three of them successfully lowered participants’ weight and/or improved physical functions. Further interventions need to focus on the prevention and control of obesity among high-risk groups, such as women and people with lower educational attainment.

